# Health behaviors of medical students decline towards residency: how could we maintain and enhance these behaviors throughout their training

**DOI:** 10.1186/s13584-021-00447-z

**Published:** 2021-04-19

**Authors:** Rachel Wilf-Miron, Ilya Kagan, Mor Saban

**Affiliations:** 1 The Gertner Institute for Epidemiology and Health Policy Research, Ramat-Gan, Israel; 2 Department of Health Promotion, School of Public Health, Sackler Faculty of Medicine, Tel Aviv University, Tel Aviv, Israel; 3 Nursing Department, School of Health Professions, Sackler Faculty of Medicine, Tel Aviv University, Tel Aviv, Israel

**Keywords:** Health behaviors, Perceived health, Emotional stress, Medical students, Physicians

## Abstract

**Background:**

We examined health behaviors and perceptions among medical students and compared them with the results of a previous survey among residents and senior physicians**.**

**Methods:**

This cross–sectional study was performed among second-year medical students (2015–2018) and among physicians (2015) using an online questionnaire. Univariate and multivariate analyses were performed.

**Results:**

Significantly more physicians perceived their health as bad, compared with students. Half of the residents, compared with one-third of senior physicians and one-fifth of students, reported high emotional stress. Residents reported the worst, and students - the best, eating habits. Logistic regression models demonstrated that lower emotional stress, healthy eating habits, adequate sleep, lower body mass index and not having a regular physician, explained good perceived health. Female gender, being a resident, bad perceived health, unhealthy eating habits, less sleep and not having a regular physician, were correlated with high emotional stress.

**Conclusions:**

The healthy lifestyle of medical students declines towards residency. Given the workload and emotional stress of their chosen profession, it is advised that medical school curriculum provide students with measures to help them to adopt healthier lifestyles, allowing students and physicians to be better role models and the healthcare system to perform better.

**Supplementary Information:**

The online version contains supplementary material available at 10.1186/s13584-021-00447-z.

## Background

Non-communicable diseases, mainly cardiovascular diseases, diabetes, cancer and chronic respiratory diseases, are leading causes of death globally [1]. Adherence to a healthy lifestyle can dramatically reduce mortality, with risk directly related to the number of unhealthy behaviors [2, 3].

Physicians are a reliable, confidential and knowledgeable source of advice on diverse health-related topics [4]. As such, they hold a unique position for promoting healthy lifestyle habits among their patients [5]. In Western countries, 65–89% of people aged 15 and over visited a physician in the primary care setting in the last year [6]. In Israel, 89% of respondents in a national survey reported seeing a primary care physician in the 12 months prior to the survey [7]. Physicians with healthy habits are more likely to discuss them with their patients, to lead an effective dialogue and to motivate their patients to adopt a healthy lifestyle [8, 9]. Physicians’ advice was perceived as more reliable if the physician disclosed his or her own health behaviors to the patient [10]. Similar findings were demonstrated among medical students [10–13].

Physician health and well-being has recently become the focus of international concern. Physician ill-health negatively affects productivity, efficiency, quality of patient care and physician retention [14], resulting in suboptimal functioning of the health system.

The Israeli healthcare system is characterized by a low hospital bed/population ratio and a high occupancy rate. Moreover, the number of practicing physicians per population is lower (with a downward trend over time) than the average of 36 Organization for Economic Co-operation and Development countries [15, 16]. As a result, practicing physicians in Israel often experience heavier workloads and difficulties maintaining a work-life balance, especially if they work in hospitals. A national survey among 4832 Israeli physicians demonstrated unfavorable health behaviors that were worse than those of the general population [17]. Residents and hospital-based physicians reported significantly less healthy lifestyles, lower perceived health and higher levels of emotional stress, compared with senior and community-based physicians [17]. Similar findings were reported for physicians in other countries [17–21].

These disturbing findings have raised the question of whether Israeli physicians have unhealthy lifestyle habits before entering medical school, or whether they adopt such unfavorable health behaviors as their training advances. To that end we examined health-related behaviors and perceptions of medical students at the beginning of their training and compared them to those of residents and senior physicians obtained using a similar survey tool during a previous national study**.**

## Material and methods

### Study design, setting and participants

This cross–sectional study was performed among students and practicing physicians (residents and senior physicians). The study protocol was approved by the Institutional Ethics Committee (Approval number 0001322–1).

The survey among medical students was conducted every year for 4 consecutive years (2015–2018). Students were recruited from one medical school, at the Tel Aviv University. Second year (preclinical) medical students studying in a 6-year program were contacted through the class’s social network (which included all registered students) during the week before the beginning of the academic year. They were asked to complete an online questionnaire. Two reminders were sent each year via the social network to the entire class.

All Israel Medical Association members were contacted by email and asked to complete an electronic questionnaire. The e-mail list of the Association comprised 95% of the country’s physicians. E-mails were sent in July 2015 with a short cover letter, containing a link to the survey. Three reminders were sent, between July and August 2015, to members who did not open the mail message.

To avoid duplication with the previous study [17], only new findings and interpretations due to additional analyses of the original data are presented.

### Questionnaire

The questionnaire was originally developed for the physician survey and was subjected to validation by an expert panel and pilot tested by 30 respondents who were asked to provide feedback on clarity, relevance and other aspects of the tool. The questionnaire comprised a section on respondent demographics and eight sections on health behaviors: (1) accumulated weekly minutes of physical activity (4 questions); (2) eating habits and nutrition (7 questions); (3) current smoking status; (4) number of hours of sleep; (5) perceived health; (6) perceived emotional stress; having a regular physician for personal care; (7) height and weight for the calculation of body mass index (BMI); and (8) personal and work characteristics (8 questions). The students’ questionnaire was identical to the physicians’ questionnaire in all topics except for 2 items: the student questionnaire had a specific question about the intensity of PA, while the physicians’ version did not. In addition, the questions on professional characteristics were different (Supplementary file 1).

### Definition of variables

The “meeting physical activity guidelines” variable, which was relevant to the students’ questionnaire only, included accumulating at least 150 min of moderate-intensity or 75 min of vigorous-intensity physical activity, in a typical week. This was calculated by multiplying the times per week that the students reported exercising by the number of minutes per exercising episode. One minute of vigorous-intensity activity is translated to 2 min of medium-intensity activity [22]. Results are presented as the medium intensity equivalent.

A composite “Healthy Nutrition” measure was defined, which included the following parameters: eating breakfast, eating lunch, adhering to a Mediterranean diet (which includes fruit and vegetables, whole wheat, legumes, nuts, fish, poultry and low-fat dairy products) [23], consuming 5 units of fruits and vegetables [24], drinking 8 glasses of water [23] – all of the above every day, or almost every day; consuming processed food or drinking sugar sweetened beverages – never or less than once a week. Respondents with healthy nutrition habits were defined as those who complied with 6 or 7 of these 7 parameters.

Perceived health was examined by the question: “In general, how would you define your health status?” (excellent, very good, good, fair, poor) [25].

Perceived emotional stress was assessed by the question: “To what extent do you experience emotional stress?” (very low, low, moderate, high, very high) [26].

### Statistical analysis

Continuous variables were summarized as mean and standard deviation. Categorical variables were summarized as number and percentage. Chi-squared test and t-test were employed for univariate analysis and comparisons among subgroups. Logistic regression models were estimated for perceived health status and stress levels, as dependent variables. A binomial variable was defined for the multivariate analysis: for perceived health status, 0 = poor or fair and 1 = good, very good or excellent. For emotional stress, 0 = very low, low or moderate and 1 = high or very high. Prior to performance of the regression, we ruled out co-linearity (in VIF test, all correlations were smaller than 2.5). We then followed the law of Hosmer and his colleagues [27] and incorporated into the model those variables which were found of statistical significance (*p* value < 0.25 in the uni-variate tests).

The level of significance for all statistical analysis was 5%. Data analysis was performed using the Statistical Package for Health & Welfare Science for Windows (SPSS, version 25.0, Chicago, IL, USA).

## Results

Among 4 consecutive classes of second-year registered medical students, 621 of 710 responded (a response rate of 87.5%). Response rates were 93.5, 86.1, 83.9 and 86.8% in the years 2015, 2016, 2017 and 2018, respectively. Of 25,590 e-mail addresses that were contacted, 14,694 physicians (57.4%) opened the email and 4832 (32.9%) completed and submitted the questionnaire. Since only half of the Israel Medical Association’ e-mails to the physicians represented by this organization are ever opened by the physicians, we calculated effective response rate as the proportion of physicians who opened the e-mail and submitted the questionnaire (32.9%). Of 4832 responding physicians, 19.5% were residents or fellows, 72.2% were senior physicians and 8% were general physicians, which is not considered a medical expertise in Israel.

Table 1 summarizes the demographics and lifestyle habits of the respondents. As expected, age was statistically significantly different among the groups of respondents due to the different stages in the physicians’ professional life cycle. There was female predominance among the students, unlike in the other groups. Half of the students reported working for more than 10 h/week in a typical week and 20.8% reported working for 20 weekly hours or more to support themselves.

**Table 1:** Participants’ demographics and lifestyle habits

Variable	Students ***N*** = 621 (%)	Residents ***N*** = 931 (%)	Seniors ***N*** = 3447 (%)	Other^**a**^ ***N*** = 396 (%)	***P*** Value
**Age, years**					
< 35	99.8	57.3	1.0	8.1	< 0.001
35–44	0.2	38.2	21.8	8.3	
45–54	–	2.6	27.0	19.7	
55–64	–	1.4	32.0	39.9	
65+	–	0.4	16.9	22.7	
Unknown	–	0.1	1.2	1.3	
**Gender**					
Male	39.6	61.7	59.4	57.1	< 0.001
**Perceived health Status**					
Excellent	20.8	11.4	11.7	6.9	< 0.001
Very good	50.7	37	34	25.2	
Good	21.8	31.9	33.7	38.2	
Fair	6.1	17.5	18.5	26.5	
Poor	0.5	2.3	2.1	3.3	
**Perceived level of emotional stress**					
Very low	6.8	4	8.1	12.8	< 0.001
Low	27.4	14.3	20.9	20	
Moderate	45.1	31.9	37.6	37.9	
High	16.7	35.1	26.1	20.8	
Very high	4.1	14.7	7.4	8.5	
**Body mass index (BMI)**^**b**^					
Underweight (< 18.5 kg/m^2^)	7.4	3.6	1.2	1.1	< 0.001
Normal weight (≥18.5 to < 25 kg/m^2^)	79.1	53.7	41.5	29	
Overweight (≥25 to < 30 kg/m^2^)	11.5	33.5	41.5	45.8	
Obese (≥30 kg/m^2^)	2.0	9.2	15.8	24.1	
**Nutrition & eating habits** (every day or almost every day)					
Breakfast	64.1	38.6	55.4	61.2	< 0.001
Lunch	73.0	46.5	48.9	51.8	< 0.001
Mediterranean Diet	40.7	25.9	35.3	40.3	< 0.001
Drinking 8 cups of water	50.1	33.7	36.1	44.1	< 0.001
Processed food	19.3	27.6	15.7	13.6	< 0.001
Sweetened beverages	11.5	18.5	9.0	12.1	< 0.001
5 units of fruits & vegetables	25.8	20.6	35.7	41.8	< 0.001
**Cigarette smoking** Currently	10.7	15.5	6.5	10.9	< 0.001
**Average hours of night sleep**					
≤ 5 Hours	8.1	43.3	20.5	22.8	< 0.001
6 Hours	25.2	43.6	53.6	51.0	
7 Hours	44.4	12.3	23.3	21.5	
≥ 8 Hours	22.3	0.8	2.6	4.7	
**Has a regular physician for personal care**	76.2	42.2	42.2	48.7	< 0.001

^a^ This entity refers to physicians who do not hold a specialty, yet they are not residents or fellows. In Israel they are referred to as “general physicians**”**

Data on professional status were missing for 65 (1.3% of practicing physicians)

^b^ Height and/or weight data for the calculation of BMI were available for 608 (99.7%) of the students; for 890 (95.6%) of the residents; for 3209 (93.1%) of the seniors and 369 (93.2%) of the “others”

Among the students, 44.6% achieved the recommended physical activity target. Among the physicians, residents reported considerably lower rates of achieving the target compared with senior physicians (15.0% vs. 34.1%). It should be noted that due to a different calculation scheme due to the absence of data on physical activity intensity in the physician group, this variable could not be compared with that of the students, probably representing an underestimation of the real proportion of physicians achieving the target.

Only 6.6% of the students perceived their health status as fair or poor, compared to 19.8% of residents, 20.6% of senior physicians and 29.8% of general physicians (*p* < 0.001).

Residents had the highest stress level: almost half of them (49.8%) reported high or very high perceived emotional stress levels, compared with 33.5% of senior physicians, 29.3% of general physicians, and 20.8% of students.

Eating habits were significantly worse among residents in all 7 items. Students demonstrated the best eating habits among the four professional groups in consuming breakfast, lunch, keeping a Mediterranean diet and drinking water. Residents demonstrated the highest rate of cigarette smoking (15.5%) compared to about 10% or less among the other respondents, and the worst sleeping habits: only 13.1% reported adequate sleep (≥ 7 hours/night) compared to 66.7% of students. Of the senior and general physicians, only 25.9 and 26.2% respectively, reported sleeping adequately. Most students (76.2%) reported having a regular physician compared to less than half of the physicians.

Multivariate analysis (Table 2) demonstrated that lower emotional stress, healthy eating habits, adequate sleep, lower BMI and not having a regular physician were predictors for good perceived health.. Being a female, younger age, professional status (with residents being mostly exposed to stress), low perceived health, unhealthy eating habits, less sleep and not having a regular physician were predictors for high emotional stress.

**Table 2:** Results of logistic regression models for the dependent variables: health and stress perceptions

Variable	B	SE	***P*** Value	OR	95% CI
**Perceived health status**					
**Gender**	−0.137	0.086	0.109	1.147	0.970–1.356
**Age**	−0.274	0.043	<0.001	0.760	0.699–0.828
**Status**					
Student	Ref group	–	–	1	–
Resident	− 0.230	0.204	0.259	0.794	0.533–1.184
Senior	−0.068	0.214	0.100	0.752	0.704–1.627
Other	−0.454	0.248	0.067	0.635	0.391–1.033
**Emotional Stress**	−0.736	0.083	< 0.001	0.479	0.407–0.564
**Nutrition**	0.206	0.024	<0.001	1.229	1.172–1.288
**Smoking**	−0.125	0.133	0.350	0.883	0.680–1.146
**BMI**	−0.113	0.009	< 0.001	0.893	0.877–0.909
**Sleep**	0.393	0.105	< 0.001	1.481	1.207–1.818
**Regular physician**	−0.343	0.082	<0.001	0.710	0.604–0.833
**Perceived level of emotional stress**					
**Gender**	−0.414	0.068	< 0.001	0.661	0.578–0.755
**Age**	−0.272	0.034	< 0.001	0.762	0.713–0.814
**Status**					
Student	Ref group	–	–	1	–
Resident	1.085	0.136	< 0.001	2.959	2.267–3.862
Senior	1.044	0.142	< 0.001	2.840	2.151–3.751
Other	0.826	0.187	< 0.001	2.285	1.583–3.300
**General health**	−0.721	0.083	< 0.001	0.486	0.413–0.572
**Nutrition**	−0.114	0.019	< 0.001	0.892	0.859–0.927
**Smoking**	0.048	0.111	0.664	1.049	0.844–1.305
**BMI**	0.000	0.008	0.949	1.000	0.985–1.014
**Sleep**	−0.556	0.081	< 0.001	0.573	0.489–0.672
**Regular physician**	−0.227	0.067	0.001	0.797	0.699–0.908

B = unstandardized regression coefficient

*BMI* body mass index, *CI* confidence interval, *OR* odds ratio, *PA* physical activity; *SE* standard error, *OR*odds ratio

Gender: male = 1, female = 0; Residency: resident = 1 senior = 0; Nutrition: six to seven items of healthy nutrition, every day or almost every day = 1; others =0; Health status: excellent, very good and good = 1; fair or poor = 0; Emotional stress: very low, low or, moderate =0; high or very high =1

## Discussion

Our study demonstrates considerable deterioration of health behaviors in the transition from medical school to practice. In particular, a significantly smaller proportion of residents, compared to students, reported favorable eating habits, adequate sleep, and having a regular physician for personal care, while a greater proportion of them reported worse perceived health status and greater stress levels compared to students.

In order to understand when health behaviors begin to decline during physicians’ professional life, it is important to understand how our sample of second-year medical students compares with medical students worldwide and with the general population. Compared with medical students from 5 schools in China, Australia and the US, our sample had a greater proportion of students who smoke, have normal BMI, and achieve target physical activity, but a smaller proportion of students who eat 5 servings of fruit and/or vegetables [13]. Compared with a large sample of Dutch preclinical medical students, less Israeli medical students achieved the recommended physical activity target, and a larger proportion smoke cigarettes [28]. Compared with second clinical-year students from the US, less Israeli students had adequate sleep (7h hours at night) [29].

In comparison to similar age groups in the general Israeli population, a greater proportion of students achieved the recommended physical activity target (30.0% vs. 44.6%) [30]. Similarly, Canadian and US medical students reported higher exercise levels compared with young adults from the general population [12]. Fewer medical students (13.5%) reported BMI values that are defined as overweight or obese compared with 36.8% of 20–44 year-olds belonging to the general Israeli population [31]. Fewer students smoke cigarettes compared with the general population with more than 12 years of education (10.7 and 17.1% respectively) [32]. More medical students reported adequate sleep compared with 20–44 year-olds in the general Israeli population (66.7% vs. 55.0%) [11, 33]. This comparison suggests that Israeli medical students have better health habits compared to the same age group in the general population.

Transition from the preclinical to the clinical years of medical school might be challenging. Examination of the health behaviors of medical students during their postgraduate or clinical years, might provide a clue as to when the decline in healthy habits begins. Increased stress, weight gain, more fast food consumption and less exercise were noted at the end of the academic year, compared with its beginning, among a small sample of Israeli medical students during the first year of their graduate (clinical) program. This was documented despite participation of the students in a 24-h intensive lifestyle curriculum delivered over 3 days [34]. In contrast, in a study conducted in the Netherlands, the health behaviors of Dutch students did not change significantly between the preclinical and clinical years of medical school [28].

The transition from medical school to residency is even more challenging. Only 6 years separate the beginning of the second (preclinical) year of medical school and the beginning of residency. The residency period is extremely demanding with long shifts, emotional burden and constant pressure on the resident to be prepared for the various activities on the ward. Consequently, many residents experience significant emotional stressors [35]. Our findings showed that a significantly smaller proportion of residents, compared to students, reported favorable eating habits, adequate sleep, and having a regular physician, while a greater proportion of them reported worse perceived health status and greater stress levels compared to students. Our findings are not unique to Israeli residents. The percentage of Internal Medicine residents in Minnesota who exercised daily decreased dramatically during residency as compared to the time before starting it (4% vs. 35%), with those meeting physical activity guidelines reporting considerably less burnout [36, 37]. Similarly, high stress levels among Swiss residents were associated with worse health and life satisfaction [38].

For many Israeli residents, who are 2–4 years older than their counterparts abroad (due to compulsory military service at the age of 18, after graduating from high school), residency is also the time for starting a family, which increases the pressure on maintaining a work-life balance. On the other hand, lack of time might not explain the huge gap between the two groups, since medical students are also exposed to intense pressure, imposed by long hours of a strict curriculum with tremendous academic demands. In addition, half of all responding students reported working for more than 10 weekly hours and 20.8% of respondents reported working 20 weekly hours or more, to support themselves.

High stress levels may lead to burnout [35]. The stress levels measured among practicing physicians, especially residents, were in accordance with the findings of the National Burnout Survey carried out in 2018, which showed that physicians had the greatest burnout from among healthcare professionals. Within the medical profession, residents were the sub-group with the highest burnout score [39].

Our study showed that being female, a younger age, resident status, unhealthy eating habits, less sleep and not having a regular physician are predictors for high stress levels. In addition, we found that healthy behaviors predicted perceived good health even more than gender or professional status.

The findings of this study should be utilized for formulating policies and practical recommendations for medical school deans and residency program managers.

Medical school students should be taught at the earliest possible stage about the importance of leading healthy lifestyles and provided with tools to help them incorporate such habits within their tight schedule. This knowledge and practical tools should be refreshed every 1–2 years. In the last decade, many medical schools have embraced a lifestyle medicine curriculum. The City University of New York School of Medicine has a 7-year program that integrates lifestyle medicine throughout the curriculum and promotes students’ healthy lifestyle on campus by two fitness centers, physical activity programs, weekly mindfulness sessions, and healthy food options [40]. In 32 US medical programs, kitchen-based nutrition education of medical students and residents was associated with healthier dietary patterns and increased their dietary counseling competencies [41]. Based on this relatively new international experience, we recommend that Israeli medical school deans establish a mandatory course to provide knowledge on lifestyle medicine to all medical students.

Healthcare administrators should be urged to invest in creating a health-promoting work environment that would encourage adapting a healthy lifestyle among healthcare staff, and particularly medical residents. For example, such a program may facilitate active transportation to work, encourage stair climbing and “walking staff meetings”. Residents (and senior physicians) should be allowed enough time for meal breaks during the workday. Healthy nutritional options should be offered at staff meetings, hospital cafeterias and in vending machines. Additionally, exercise and interventions for reducing stress and increasing the quality of life should be encouraged. A team-based voluntary exercise program increased the quality of life and decreased burnout rates among residents and fellows, while self-care workshops and meditation interventions reduced stress and burnout [37, 42]. Internal medicine residents who achieved physical activity targets reported considerably less burnout [36, 37]. However, offering health-promoting programs is not sufficient since physicians must move forward to utilize them. According to a 2017 National Physician Health Survey, carried out by the Canadian Medical Association, 81% of physicians and residents said they were aware of physician health program services available to them, yet only 15% had accessed them [43]. Such a gap between awareness and implementation, and the magnitude of the problem portrayed in our study, emphasize the importance of accompanying practical recommendations with policy formulation. In 2017, The American Medical Association House of Delegates released Resolution 959 (I-17), supporting policies and mechanisms that incentivize or provide funding for the inclusion of lifestyle medicine education in undergraduate, graduate and continuing medical education [44]. It seems that new policies are also required in Israel to transform the current curriculum and the medical work environment across the continuum of medical education. A collaboration among the Ministry of Health, the Israel Medical Association, deans of medical schools and managers of residency programs is highly recommended to formulate and implement such policies and design the appropriate mechanisms to evaluate their effect on health and wellness of medical students and residents.

Designing a work environment and culture that promote health and wellness during all stages of professional training – from medical school, into residency and onto senior physician life - would benefit not only physicians, but also other health professionals who work in the same environment, as well as patients, their families and the healthcare system at large.

This study has several limitations: First, because the student and physician surveys had a different definition to achieving the target of recommended physical activity, it was not possible to compare this important health behavior between the students and the physicians, and therefore it was not included in the multivariate analysis. It may be assumed that achieving the physical activity target would have been found to be protective against emotional stress, as was demonstrated among practicing physicians [12]. Second, medical students’ behaviors were collected by a cross-sectional survey during 4 consecutive years. Since health behaviors seems to decline between the beginning of medical school and residency, it is important to understand when this decline starts. A longitudinal cohort study among medical students, with the survey taken at various times during the preclinical and clinical years could provide a clue and help in planning appropriate interventions. Despite this limitation, our study is unique in the fact that it uses the same survey tool, in a similar time period and in the same, relatively small country, to compare health behaviors of students and practicing physicians. A third limitation refers to the fact that while asking about eating 5 units of fruits and/or vegetables per day, the questionnaire did not explain what “a unit” means, what might result in different interpretations of the term “unit” by responders. The fourth limitation stems from the different recruiting of the study participants: Medical students were recruited from one medical school in central Israel while the physicians were recruited from all over Israel. This might influence the proportion of ethnic minorities or respondents living in the geographic periphery, both having a possible impact on the health behaviors and self-rated health. The last limitation refers to the validation of the questionnaire. In further research of student and physician’s health behaviors, it is recommended to validate the questionnaire, using a construct validity.

## Conclusions

This study demonstrated that at the beginning of medical school, students lead a healthy lifestyle, which declines over time and is less healthy during their residency. Given the workload and emotional stress of their chosen profession, medical school curriculum should provide students with tools and skills to help maintain health habits throughout their professional life, to protect them from work-related health risks and allow them to gain more years of healthy life. In addition to the invaluable contribution to students’ and practicing physicians’ healthy lifestyles, such efforts would allow the healthcare system to perform better and for physicians to be better role models for their patients.

## Supplementary Information

**Additional file 1.** English translation of the study questionnaire.

## Data Availability

The data that support the findings of this study are available from the corresponding author, upon reasonable request.

## Abbreviations

BMI: Body mass index
