# Supplementary material for: Health behaviors of medical students decline towards residency: how could we maintain and enhance these behaviors throughout their training
Source: Isr J Health Policy Res. 2021 Apr 19;10:13. doi: 10.1186/s13584-021-00447-z (PMC8054363; doi:10.1186/s13584-021-00447-z)
Supplement: Supplementary file 1 — Additional file 1. English translation of the study questionnaire. [file 13584_2021_447_MOESM1_ESM.docx]

**Additional file 1: English translation of the study questionnaire**

**Do medical students lead a healthy lifestyle?**

Dear students

Just before we begin the course: “Promoting a healthy lifestyle – the physician' role”, I kindly ask you to dedicate 2 minutes for completing a short questionnaire. Your privacy and confidentiality are guaranteed. The survey findings will be reflected to the class during the first session of the course.

Sincerely yours

Course coordinator

1. **In a typical week, how many times do you perform moderate-intensity physical activity** such as brisk walking, body-building exercises, ball games, dance or yoga?

- Not at all
- 1
- 2
- 3
- 4
- 5
- 6
- 7

1. **On average, how long does each episode of activity last?** ______ minutes
2. **In a typical week, how many times do you perform vigorous-intensity physical activity** that causes considerable increase in respiratory and heart rates as well as profound sweating, such as running, swimming or rapid cycling?

- Not at all
- 1
- 2
- 3
- 4
- 5
- 6
- 7

1. **On average, how long does each episode of activity last?** ______ minutes

**Which of the following questions (numbers 5-11) best describes your nutrition and eating habits?**

1. **Eating breakfast**

- Daily or almost daily
- 3-4 times a week
- 1-2 times a week
- Less than once a week
- Not at all

1. **Eating lunch during the working day**

- Daily or almost daily
- 3-4 times a week
- 1-2 times a week
- Less than once a week
- Not at all

1. **Adherence to the principles of a Mediterranean diet**: intake of vegetables, fruit, legumes, olive oil, nuts, unrefined wheat, fish, poultry and low-fat dairy products.

- Daily or almost daily
- 3-4 times a week
- 1-2 times a week
- Less than once a week
- Not at all

1. **Drinking 8 cups of water per day**

- Daily or almost daily
- 3-4 times a week
- 1-2 times a week
- Less than once a week
- Not at all

1. **Eating processed food products**

- Daily or almost daily
- 3-4 times a week
- 1-2 times a week
- Less than once a week
- Not at all

1. **Drinking sugar-sweetened beverages, including fruit juices**

- Daily or almost daily
- 3-4 times a week
- 1-2 times a week
- Less than once a week
- Not at all

1. **Eating 5 units of fruits and/or vegetables per day**

- Daily or almost daily
- 3-4 times a week
- 1-2 times a week
- Less than once a week
- Not at all

1. **At present, do you smoke cigarettes?**

- Yes
- No

1. **In the last month, how many hours, on average, did you sleep at night?**

- 5 or less
- 6
- 7
- 8 or more

1. **In general, how would you define your health status?**

- Excellent
- Very good
- Good
- Fair
- Poor

1. **To what extent do you experience emotional stress?**

- Very low
- Low
- Moderate
- High
- Very high

1. **Do you have a regular physician for your personal health?**

- Yes
- No

1. **Your gender**

- Male
- Female

1. **Your age**

- <35
- 35-44
- 45-54
- 55-64
- 65+

1. **For the calculation of body mass index (BMI)**

- Your height in centimeters is_____ (according to the last measurement that you can recall)
- Your weight in kilograms is______ (according to the last time you stood on the scales)

1. **How many hours do you work each week (extra-curricular work) during the academic year?**

- Do not work during the year
- Up to 10 hours
- 11-20 hours
- 21-30 hours
- More than 30 hours

**Thank you for your cooperation**
